# Efficacy of *Lycium barbarum L.* on plasma lipid concentration in adults: A protocol for systematic review and meta-analysis

**DOI:** 10.1097/MD.0000000000028172

**Published:** 2021-12-10

**Authors:** Xueyuan Zeng, Weimin Zhao, Yunlong Xu, Chengwei Zhang, Junliang Wu, Libo Xia, Ziyue Tian, Jixiang Ren

**Affiliations:** a Chinese Medicine Academy, Changchun University of Chinese Medicine, Changchun, Jilin Province, PR China; b Department of Prevention, the Affiliated Hospital to Changchun University of Chinese Medicine, Changchun, Jilin Province, PR China.

**Keywords:** *Lycium barbarum L*, meta-analysis, plasma lipid concentration, protocol

## Abstract

**Background:**

Dyslipidemia is an important risk factor for atherosclerotic cardiovascular disease. *Lycium barbarum L.* are widely used as medicinal and functional food and may be particularly beneficial for patients with dyslipidemia. This systematic review protocol is designed to be used to evaluate the effects of *Lycium barbarum L.* on plasma lipid concentration through systematic reviews and meta-analysis.

**Methods:**

The Following electronic databases will be searched from inception to October 2021: the China National Knowledge Infrastructure, PubMed, Cochrane Library, Web of Science, and Wan-fang database. All randomized controlled trial designs evaluated the effects of *Lycium barbarum L.* on plasma concentrations of lipids will be included. Two researchers will operate literature retrieval, screening, information extraction, quality assessment, and data analysis independently. The analysis will be conducted using Rstudio software (Version 1.4.1717).

**Results:**

The findings will be submitted to a peer-reviewed publication.

**Conclusion:**

This study will provide practical and targeted evidence in investigating the impact of *Lycium barbarum L*. on plasma lipid concentration in adults.

**Registration number:**

INPLASY2021110043

## 1 Introduction

Dyslipidemia refers to a group of lipid abnormalities including elevated low-density lipoprotein cholesterol (LDL-C), hypercholesterolemia, hypertriglyceridemia, and low high-density lipoprotein cholesterol (HDL-C).^[1]^ Dyslipidemia is an important risk factor for atherosclerotic cardiovascular disease.^[2]^ Many studies have shown that lowering LDL-C levels can significantly reduce the risk of morbidity and mortality from atherosclerotic cardiovascular disease.^[3,4]^ Prevention and proper management of dyslipidemia can significantly change cardiovascular morbidity and mortality. However, the management of dyslipidemia is extremely challenging. Statins are the cornerstone of drug therapy for dyslipidemia. However, statins may cause some adverse reactions, such as elevated liver transaminase, muscle damage, elevated blood sugar, and cognitive impairment.^[5]^ Diet and lifestyle are one of the important causes of dyslipidemia. Diet treatment and lifestyle improvement are the basic measures to treat dyslipidemia.^[6]^

In recent years, more and more natural medicines have been found to have active ingredients to improve dyslipidemia. The intervention of Chinese medicine on dyslipidemia has aroused people's attention. Among them, Chinese wolfberry also known as *Lycium barbarum L.* or Goji berry, is widely used in the Asia-Pacific region as both medicine and food. *Lycium barbarum L.* were first used as medicinal plants about 2300 years ago.^[7,8]^ In China, it is used as traditional Chinese medicine and functional food in daily life because of its health benefits. *Lycium barbarum L.* has the effect of nourishing liver and tonifying kidney, protecting eyesight. Modern medical research shows that wolfberry has anti-aging, anti-oxidation, anti-diabetes, anti-cancer, cell protection, neuroprotection, and immunomodulatory effects.^[9–14]^ Using chromatographic techniques, the researchers identified a variety of chemical components, including polysaccharides, carotenoids, flavonoids, and alkaloids. L. barbarum polysaccharides was the most widely studied. L. barbarum polysaccharides has a variety of physiological effects, such as antioxidant function, antitumor activity, neuroprotection, immunomodulatory function, and other biological functions.^[15]^

In in vitro and in vivo studies, *Lycium barbarum L.* exhibited lipid-lowering, antioxidant, and anti-inflammatory properties.^[16,17]^ However, the results of randomized controlled trials on lycium barbarum *L.* have been inconsistent in clinical studies. Studies have shown that taking goji berries for 45 days on a healthy diet can significantly improve lipoprotein levels in patients with metabolic syndrome.^[18]^ Using standardized goji berry juice can improve neuropsychological performance, as well as overall health and well-being.^[19]^ In contrast, other studies indicated that standardized goji berry juice or Wolfberry have no benefits on weight, blood pressure, and blood lipids.^[20–23]^ The effect of *Lycium barbarum L.* supplementation on lipid concentration is controversial. Whether the hypolipidemic effect of Wolfberry is consistent in healthy people or clinical population is considered. Therefore, we suggest a systematic review and meta-analysis of the effects of *Lycium barbarum L.* on plasma lipid concentration.

## 2 Methods

### 2.1 Study registration

The protocol for this systematic review was registered on INPLASY (registration number INPLASY2021110043). Our review will comply with the guidelines of the Preferred Reporting Items for Systematic Reviews and Meta-Analyses (PRISMA) statement guidelines.^[24]^

### 2.2 Inclusion criteria for study selection

#### 2.2.1 Inclusion/exclusion of articles

Trials with the following inclusion criteria will be included: applied a RCT; evaluated the effects of *Lycium barbarum L.* on plasma/serum concentrations of lipids will be included; and presented mean and standard deviation (SD) in both intervention and control groups. Due to language restrictions, we will search for articles in English and Chinese to get a more objective and a true evaluation.

#### 2.2.2 Type of participants

Subjects aged over 18 will be included in the review. There will be no restriction on sex, ethnicity, or disease duration.

#### 2.2.3 Type of intervention

Comparison interventions included placebo control, no therapy, exercise intervention, diet intervention, or active pharmacological treatment. Studies that administrated *Lycium barbarum L.* in combination with other components will be excluded.

#### 2.2.4 Type of comparators

Studies for the effect of *Lycium barbarum L.* on plasma lipid concentration in adults will be included. The only difference between the control and treatment groups was *Lycium barbarum L.*

#### 2.2.5 Outcome measurements

The primary aim is to review the literature and synthesize relevant data to investigate the effect***s*** of *Lycium barbarum L.* on plasma concentrations of lipids in adults comprehensively. Variables such as the concentrations of total cholesterol (TC), triglyceride (TG), LDL-C, and HDL-C will be included.

### 2.3 Search strategy

The following electronic databases will be searched from inception to October 2021: the China National Knowledge Infrastructure, PubMed, Cochrane Library, Web of Science, and Wan-fang databases. The search was restricted to published studies on humans without language restriction. We used the following keywords as search terms: (“Lycium barbarum L.” OR “L. barbarum” OR “ Goji” OR “lyceum” OR“ wolfberry”) AND (“Intervention Studies” OR “intervention” OR “controlled trial” OR “randomized” OR “randomised” OR “random” OR “randomly” OR “placebo” OR “assignment”). About other sources, we also plan to manually search for the unpublished conference articles and the bibliography of established publications. In addition, a manual search of recent reviews, meta-analyses, and original studies were also scrutinized.

### 2.4 Data collection and analysis

#### 2.4.1 Selection of studies

**Figure 1:**
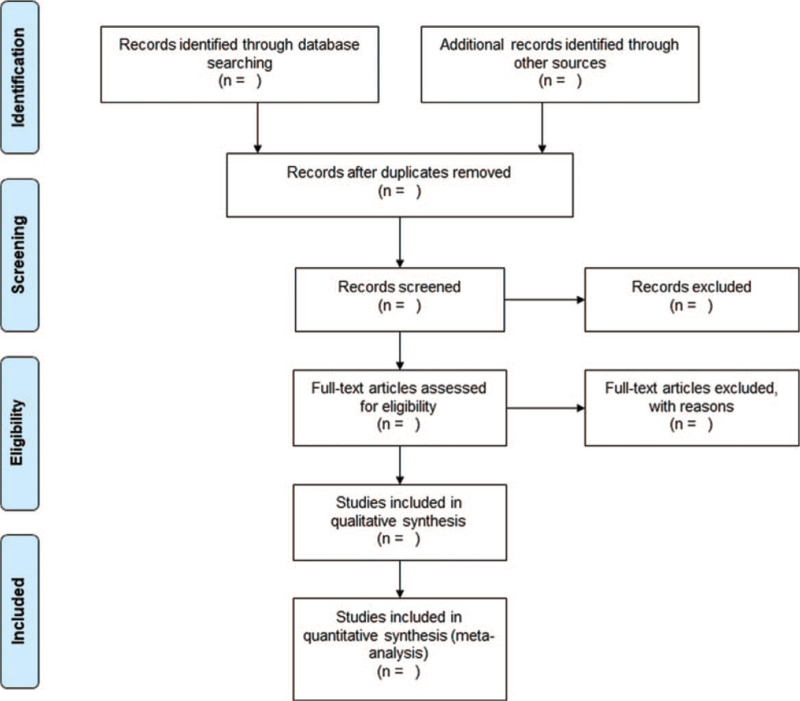
The preferred reporting items for systematic review and meta-analysis flow diagram.

A PRISMA flow diagram will be used to document the whole trial selection process (Fig. 1). Studies retrieved in the databases will be uploaded to the software reference manager EndNote X8 for screening. Abstracts of all articles will be screened independently by 2 reviewers based on eligibility criteria. If there is any inconsistency in the inclusion of an article, a third reviewer will be consulted independently. Retained articles will be reviewed in full.

#### 2.4.2 Data extraction and management

The authors will follow the inclusion criteria strictly and selected RCT articles related to the topic. Data for the trials will be extracted independently by 2 review authors using a standard form. The extracted information was as follows: study characteristics (first author's last name, year of publication, location of the study, sample size, and study design); participants’ information (gender, mean age, mean body mass index, and health status); intervention details (duration of treatment and dose); and investigated outcomes including TC, TG, LDL-C, and HDL-C.

#### 2.4.3 Assessment of bias in the included studies

Each trial included in the review will be independently assessed by 2 reviewers for potential biases. For any disagreement, a third reviewer will help to confirm the final assessment. Original article authors will be contacted (when possible), if there are insufficient details to confidently assess the risk of bias. We will use the Cochrane collaborative tool to independently assess the risk of bias in the included studies. The Cochrane Collaboration's tool assigns scores for following domains: random sequence generation (selection bias); allocation concealment (selection bias); blinding of participants and personnel (performance bias); blinding of outcome assessment (detection bias); incomplete outcome data (attrition bias); selective reporting (reporting bias); and other sources of bias. The risk of bias is evaluated at 3 levels, namely, low risk, high risk. A risk of bias summary table will be produced.

#### 2.4.4 Data analysis

When the data permits, the analysis will be done using Rstudio software (Version 1.4.1717). Preintervention and postintervention means ± SDs of TC, TG, LDL-C, and HDL-C in both *Lycium barbarum L.* and control groups will be extracted. All of standard error of mean was changed to SD by multiplying standard error of mean to √n. We used the suitable formula (SD_change_ = square root [(SD_baseline_^2^ + SD_final_^2^) − (2 × r × SD_baseline_ × SD_final_)]) to calculate SD of the mean difference in studies that did not report this parameter. Correlation coefficient will be estimated according to following formula: r = (SD_1_^2^ + SD_2_^2^ − SD_Change_^2^)/2SD_1_ × SD_2._ The heterogeneity between the included studies will be analyzed by Cochran Q test and Higgins I^2^ statistic. If there is no statistical heterogeneity between the results of each study (*P* > .1, I^2^<50%), the fixed-effect model will be used for meta-analysis. If not, the source of heterogeneity will be further analyzed and the random-effect model will be used for analysis after excluding the influence of obvious clinical heterogeneity. All outcomes will be analyzed through 95% confidence intervals. If there are insufficient data to support meta-analysis, descriptive analysis will be implemented.

#### 2.4.5 Subgroup analysis

Subgroup analyses will be conducted for based on gender, intervention duration, *Lycium barbarum L*. dose, and type of study population (healthy or unhealthy) when data are available.

#### 2.4.6 Sensitivity analysis

Sensitivity analysis is performed when Cochran Q test and Higgins I^2^ statistic show significant heterogeneity. We will eliminate each study one by one to identify literature that has apparent impact on the original effects. The reasons for the obvious heterogeneity of the literature will be scrutinized from the aspects of sample size, study quality, and statistical methods.

#### 2.4.7 Evidence assessed

The evidence quality will be evaluated by the recommended grading method of the GRADE system, and the evidence quality is divided into 4 levels, namely A (high quality), B (medium quality), C (low quality), and D (very low quality).

#### 2.4.8 Assessment of reporting biases

If more than 10 articles will be included, a funnel plot would be used to demonstrate publication bias by its symmetry.

#### 2.4.9 Ethics and dissemination

Because this study does not involve private patient information, no ethical review is required. The results of this system review and meta-analysis will be published in a peer-reviewed journal.

## 3 Conclusion

This review protocol was used for a systematic review and meta-analysis to determine the effect of *Lycium barbarum L*. on plasma lipid concentrations. It clearly describes the type of trial, participants, interventions and results, as well as search strategies, data sources, extraction methods, and data analysis. Our search strategy will include both published and unpublished studies. Dyslipidemia, as an important risk factor of cardiovascular disease, has attracted wide attention in the world. Whether *Lycium barbarum L*. has lipid-lowering effects will be verified in our systematic review and meta-analysis. We hope this study will provide evidence for the intervention of *Lycium barbarum L*. in lipid therapy and guide future research plans.

## Author contributions

**Conceptualization:** Xueyuan Zeng, Jixiang Ren, Weimin Zhao

**Data curation:** Xueyuan Zeng, Libo Xia, Junliang Wu

**Formal analysis:** Xueyuan Zeng, Yunlong Xu, Chengwei Zhang

**Funding acquisition:** Jixiang Ren

**Methodology:** Xueyuan Zeng, Jixiang Ren

**Project administration:** Weimin Zhao, Jixiang Ren

**Resources:** Junliang Wu, Chengwei Zhang, Ziyue Tian

**Software:** Xueyuan Zeng, Yunlong Xu

**Supervision:** Jixiang Ren, Xueyuan Zeng

**Writing – original draft:** Xueyuan Zeng

**Writing – review & editing:** Xueyuan Zeng, Jixiang Ren, Weimin Zhao
